# Association of hemoglobin, albumin, lymphocyte, and platelet score with risk of cerebrovascular, cardiovascular, and all-cause mortality in the general population: results from the NHANES 1999-2018

**DOI:** 10.3389/fendo.2023.1173399

**Published:** 2023-06-23

**Authors:** Hong Pan, Shasha Lin

**Affiliations:** ^1^ Department of Neurology, Deqing People’s Hospital (Deqing Campus, Sir Run Run Shaw Hospital, School of Medicine, Zhejiang University), Huzhou, China; ^2^ Department of Ultrasound, The First Affiliated Hospital of Wenzhou Medical University, Wenzhou, China

**Keywords:** HALP score, cerebrovascular mortality, cardiovascular mortality, all-cause mortality, NHANES

## Abstract

**Background and aims:**

Cardiovascular and cerebrovascular disease (CCDs) contribute to leading causes of morbidity and mortality in the United States of America (USA). Hemoglobin, albumin, lymphocyte, and platelet (HALP) score, a simple and convenient indicator, could reflect the combination of inflammation and nutritional status. This study was undertaken to evaluate the associations between HALP score and risk of cardiovascular, cerebrovascular, and all-cause mortality in the general population from the National Health and Nutrition Examination Survey (NHANES) 1999–2018.

**Methods:**

We identified 21,578 participants during the 1999-2018 cycles of the NHANES in this research. HALP score was calculated as hemoglobin (g/L) × albumin (g/L) × lymphocytes (/L)/platelets (/L). Outcomes were cerebrovascular, cardiovascular, and all-cause mortality determined by the NHANES-linked National Death Index record and followed until 31 December 2019. Survey-weighted Cox regression, restricted cubic spline analysis, and subgroup analysis were applied to investigate relationships between HALP score and risk of mortality.

**Results:**

This cohort study comprised 49.2% male and 50.8% female, of which the median age was 47 years old. In multivariate survey-weighted Cox regression adjusting for all confounders, compared with participants with low HALP scores, participants with highest HALP score had a lower risk of all-cause mortality (adjusted HR:0.80, 95% CI: 0.73, 0.89, *P* < 0.0001) and cardiovascular mortality (adjusted HR:0.61, 95% CI: 0.50, 0.75, *P* < 0.0001), and mediate HALP score had the lowest risk of all-cause mortality (adjusted HR:0.68, 95% CI: 0.62, 0.75, *P* < 0.0001) and cardiovascular mortality (adjusted HR:0.60, 95% CI: 0.48, 0.75, *P* < 0.0001). Restricted cubic spline analysis showed a non-linear relationship between HALP score and cardiovascular and all-cause mortality (all *P* values <0.001).

**Conclusion:**

HALP score was independently associated with risk of cardiovascular and all-cause mortality, but not cerebrovascular mortality.

## Introduction

Cardiovascular and cerebrovascular diseases (CCDs), referring to ischemic or hemorrhagic diseases of the heart, brain, and systemic tissues, are two leading causes of disability and mortality worldwide (1, 2). Data from World Health Organization indicated that over 17 million people suffered from cardiovascular or cerebrovascular mortality in 2015, covering 31% of all global deaths (3). In addition, CCDs impose a heavy burden on the economies of countries, with 18% of disability-adjusted life years lost in high-income countries, and 10% in low-income and middle-income countries (4). Therefore, identifying modifiable factors to predict reliably in those suffering from cardiovascular and cerebrovascular mortality is of great clinical relevance.

Many factors were found in the development of CCDs, such as genetics, lifestyle, dietary behavior, age, secondary behavior, and cardiometabolic diseases, and in most of these processes, inflammation can be seen (5). Anemia and thrombosis could exacerbate inflammation, while lymphocytes reduce inflammation (6). Serum albumin plays an important role in reflecting nutritional status. Some studies have also considered albumin as an indicator of the severity of inflammation and illness in acute diseases (7). Hemoglobin, albumin, lymphocyte, and platelet (HALP) score is a simple and convenient indicator, which requires only complete blood count and albumin value, reflecting the combination of inflammation and nutritional status. Since its appearance, HALP has gained a wide range of attention as a new biomarker to predict several clinical outcomes in a variety of diseases. Compelling evidence showed that HALP score is a good predictor of mortality, especially in stomach, bladder, prostate, and kidney cancers and patients with stroke (8–11).

In the last several years, although HALP has emerged in the literature as a new prognostic biomarker in various diseases, study regarding the impact of HALP score on the risk of specific and all-cause mortality in the general population is limited. To address the research gap, we conducted the present study to estimate the associations of HALP score with risk of long-term cerebrovascular, cardiovascular, and all-cause mortality in general populations.

## Methods

### Study population

**Figure 1:**
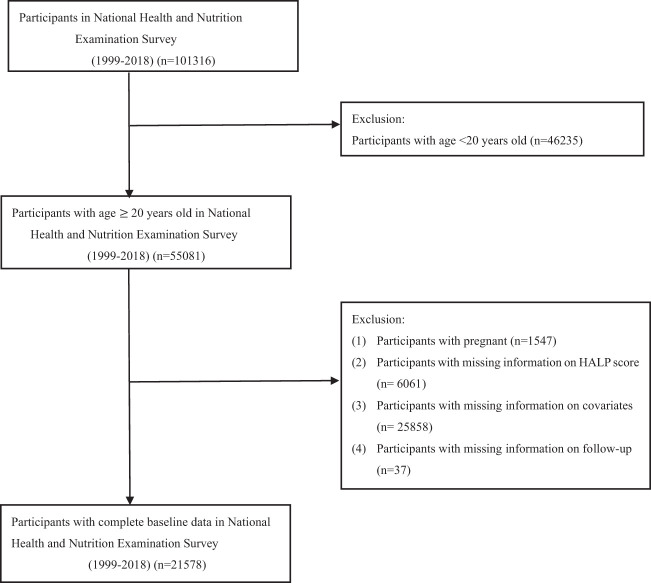
Flow chart of study population inclusion and exclusion.

This cohort study extracted 101,316 participants from the 1999-2018 cycles of the NHANES, which was conducted by the National Center for Health Statistics (NCHS) to evaluate the overall nutrition and health status of the US population. Exclusion criteria were younger < 20 years of age, pregnancy, missing information on HALP score or covariates, and loss to follow up. Collectively, 21,578 participants were enrolled in this study (**Figure 1**).

NHANES research has been approved by the Ethics Review Committee of the National Center for Health Statistics (NCHS) Research Ethics Review Board. All participants signed the informed consent form at the time of recruitment in this survey.

### Definition of hemoglobin, albumin, lymphocyte, platelet score

Blood specimens were conducted according to established venipuncture protocol and procedures. Hemoglobin, lymphocyte, and platelet values were measured by hematology-analyzing device (UniCel DxH 800 Analyzer) and serum albumin levels by Roche modular P and Roche Cobas 6000 chemistry analyzers. HALP score was calculated as hemoglobin (g/L) × albumin (g/L) × lymphocytes (/L)/platelets (/L).

### Assessment of covariates

Variables used in this study included age, sex (male, female), race (White, Black, Mexican, other), educational status (<9th, 9-11th, high school, college or above), and smoking status (never, former, current). Diabetes was identified by self-reported diabetes, use of diabetes medication or insulin, glycated hemoglobin A1c (HbA1c) ≥6.5%, fasting glucose ≥126 mg/dL (7.0 mmol/L), or oral glucose tolerance test 2-h glucose ≥200 mg/dL (11.1 mmol/L). Hypertension was diagnosed by self-reported hypertension, taking medicines of anti-hypertension drugs, systolic pressure higher than 140mm/Hg, or diastolic pressure higher than 90mm/Hg at least measured three times. Hyperlipidemia was diagnosed as total cholesterol (TC) level ≥200 mg/dL (≥5.18 mmol/L); triglycerides level (TG) level ≥150 mg/dL; low density lipoprotein-cholesterol (LDL-c) value ≥130 mg/dL (≥3.37 mmol/L); High-density lipoprotein-cholesterol (HDL-c) level < 40 mg/dL (1.04 mmol/L) in men and 50 mg/dL (1.30mmol/L) in women; or use of anti-hyperlipidemia drugs. Body mass index (BMI) was measured as weight (kilograms) divided by height (meters) squared. Besides, laboratory indicators such as TC (total cholesterol), TG (triglycerides), LDL-C (low-Density Lipoprotein Cholesterol), HDL-C (high-Density Lipoprotein Cholesterol), and HbA1c (glycated hemoglobin) were also obtained as covariates in this analysis.

### Ascertainment of outcomes

Endpoints of this study were cerebrovascular, cardiovascular, and all-cause mortality, which were extracted from NHANES-linked National Death Index record (NDI). Cerebrovascular mortality was defined as I60–I69, and cardiovascular mortality was defined as I00-I09, I11, I13, I20-I51 according to International Classification of Diseases, 10^th^ Revision (ICD-10) (12).

### Statistical analysis

To ensure representativeness of the sample in the US population, all analyses included oversampling, clustering, and stratification in this study (13). HALP score were categorized into three tertiles (<36, 36-48, >48), and group of <36 was defined as reference group. Continuous variables were expressed as weighted means and 95% confidence interval (CI), and categorical variables as weighted percentages. The weighted Kruskal Wallis tests for continuous variables and weighted Chi-square test for categorical variables were applied to compare the intergroup difference. Associations of tertiles of HALP score with risk of cerebrovascular, cardiovascular, and all-cause mortality were estimated by weighted Cox proportional hazard regression models. Three models of Cox proportional hazard regression were established as follows: model 1 was a crude model without adjusting other covariate, model 2 was adjusted for partial variables (age, sex, race, education, and smoke), and model 3 was adjusted for all variables. Furthermore, restricted cubic spline regression was used to explore relationships between HALP score and risk of cerebrovascular, cardiovascular, and all-cause mortality. Subgroup analyses stratified by age (<60 and ≥60 years), sex (male and female), race (White, Black, Mexican, and other), smoking status (former, never, and now), education (<9th, 9-11th, high school, and college or above), hypertension (yes and no), DM (yes and no), hyperlipidemia (yes and no), and BMI (<25, 25-29, ≥30) were conducted to test potential interaction effects.

All analysis were performed with R (version 4.1.2, R Foundation for Statistical Computing, Vienna, Austria), and *p* < 0.05 was set as a significant level in this study.

## Results

### Baseline characteristics

A total of 101,316 participants were initially enrolled. Following the exclusion, 21,578 adult participants in NHANES from 1999 to 2018 were finally enrolled in our analysis (**Figure 1**). This cohort study comprised 49.2% male and 50.8% female, with median age of 47 years old. All baseline covariates had significant differences (all *P* values < 0.05) among groups of HALP score, except for covariates of BMI, hyperlipidemia, and cerebrovascular disease mortality. During a median follow-up of 109 months, 3258 (15.1%) participants experienced all-cause mortality, 835 (3.9%) participants suffered from cardiovascular disease mortality, and 180 (0.8%) participants died from cerebrovascular disease mortality. Demographic characteristics of the study participants were presented in **Table 1**.

**Table 1:** Baseline characteristics of different HALP score groups among study population.

Variable	Total	Tertil 1 < 40	Tertil 2 40-56	Tertil 3 > 56	*P* value
Age, year	47.0 (34.0,60.0)	50.0 (38.0,63.0)	46.0 (33.0,59.0)	44.0 (31.0,58.0)	< 0.0001
BMI, kg/m2	27.6 (23.9,32.0)	27.4 (23.8,32.3)	27.4 (23.9,32.0)	27.8 (24.1,31.8)	0.5100
Sex, n (%)					< 0.0001
Male	10616 (49.2)	2242 (30.3)	3456 (46.9)	4918 (67.6)	
Female	10962 (50.8)	4668 (69.7)	3823 (53.2)	2471 (32.4)	
Race, n (%)					< 0.0001
White	9684 (44.9)	3196 (69.9)	3342 (70.3)	3146 (67.2)	
Black	4293 (19.9)	1666 (13.2)	1266 (8.9)	1361 (9.6)	
Mexican	3747 (17.4)	1007 (6.4)	1325 (8.2)	1415 (9.5)	
other	3854 (17.9)	1041 (10.5)	1346 (12.5)	1467 (13.7)	
Education, n (%)					< 0.0001
<9^th^	2609 (12.1)	772 (5.8)	877 (6.0)	960 (6.5)	
9-11^th^	3190 (14.8)	971 (10.2)	1013 (10.7)	1206 (12.8)	
High school	4940 (22.9)	1562 (23.1)	1614 (23.5)	1764 (25.4)	
College or above	10839 (50.2)	3605 (60.9)	3775 (59.7)	3459 (55.3)	
Smoke, n (%)					< 0.0001
Never	11627 (53.9)	4041 (58.5)	4060 (54.5)	3526 (47.0)	
Former	5467 (25.3)	1876 (27.4)	1815 (25.1)	1776 (24.4)	
Now	4484 (20.8)	993 (14.1)	1404 (20.4)	2087 (28.6)	< 0.0001
HB (g/L)	14.4 (13.5,15.4)	13.7 (12.8,14.6)	14.4 (13.6,15.3)	15.1 (14.3,16.0)	< 0.0001
Albumin (g/L)	43.0 (40.0,45.0)	41.0 (39.0,43.0)	43.0 (41.0,45.0)	44.0 (42.0,46.0)	< 0.0001
Lym (/L)	1.9 (1.5,2.3)	1.5 (1.2,1.8)	1.9 (1.6,2.2)	2.3 (2.0,2.7)	< 0.0001
PLT (/L)	242 (206.0,286.0)	279.0 (237.0,325.0)	245.0 (213.0,282.0)	213.0 (183.0,248.0)	
TC, mg/dl	191.0 (166.0,218.0)	193.0 (167.0,220.0)	192.0 (168.0,219.0)	189.0 (164.0,217.0)	< 0.001
TG, mg/dl	104.0 (72.0,152.0)	97.0 (69.0,139.0)	102.0 (71.0,151.0)	113.0 (78.0,165.0)	< 0.0001
LDL-C, mg/dl	113.0 (91.0,137)	112.00 (91.0,136.0)	114.0 (92.0,137.0)	113.0 (90.0,138.0)	0.0400
HDL-C, mg/dl	51.0 (43.0,63.0)	55.0 (45.0,67.0)	52.0 (43.0,63.0)	48.0 (40.0,58.0)	< 0.0001
HbA1c, %	5.4 (5.1,5.7)	5.4 (5.2,5.7)	5.4 (5.1,5.7)	5.4 (5.10,5.70)	0.0400
Diabetes, n (%)					< 0.0001
Yes	4071 (18.9)	1313 (14.1)	1294 (13.0)	1464 (15.2)	
No	17507 (81.1)	5597 (85.9)	5985 (87.0)	5925 (84.9)	
Hypertension, n (%)					< 0.0001
Yes	9356 (43.4)	3288 (41.2)	3007 (35.7)	3061 (37.0)	
No	12222 (56.6)	3622 (58.8)	4272 (64.3)	4328 (63.0)	
Hyperlipidemia, n (%)					0.9600
Yes	15679 (72.6)	4998 (71.7)	5288 (71.4)	5393 (71.6)	
No	5899 (27.3)	1912 (28.3)	1991 (28.6)	1996 (28.4)	
Outcomes, n (%)					
Cardiovascular disease mortality	835 (3.9)	377 (3.9)	234 (2.1)	224 (2.1)	< 0.0001
Cerebrovascular disease mortality	180 (0.8)	60 (0.6)	62 (0.5)	58 (0.5)	0.9700
All-cause mortality	3258 (15.1)	1376 (14.6)	933 (8.7)	949 (9.5)	< 0.0001

Weighted mean for continuous variables: p-value was calculated by weighted Kruskal-Wallis test. Numbers (unweighted) and percentages (weighted) for categorical variables: p-value was calculated by weighted chi-square test.

BMI, body mass index; HB, hemoglobin; Lym, lymphocyte; PLT, platelet; TC, total cholesterol; TG, triglycerides; LDL-C, low-Density Lipoprotein Cholesterol; HDL-C, high-Density Lipoprotein Cholesterol; HbA1c, glycated hemoglobin.

### Associations of HALP score with risk of long-term mortality

**Figure 2:**
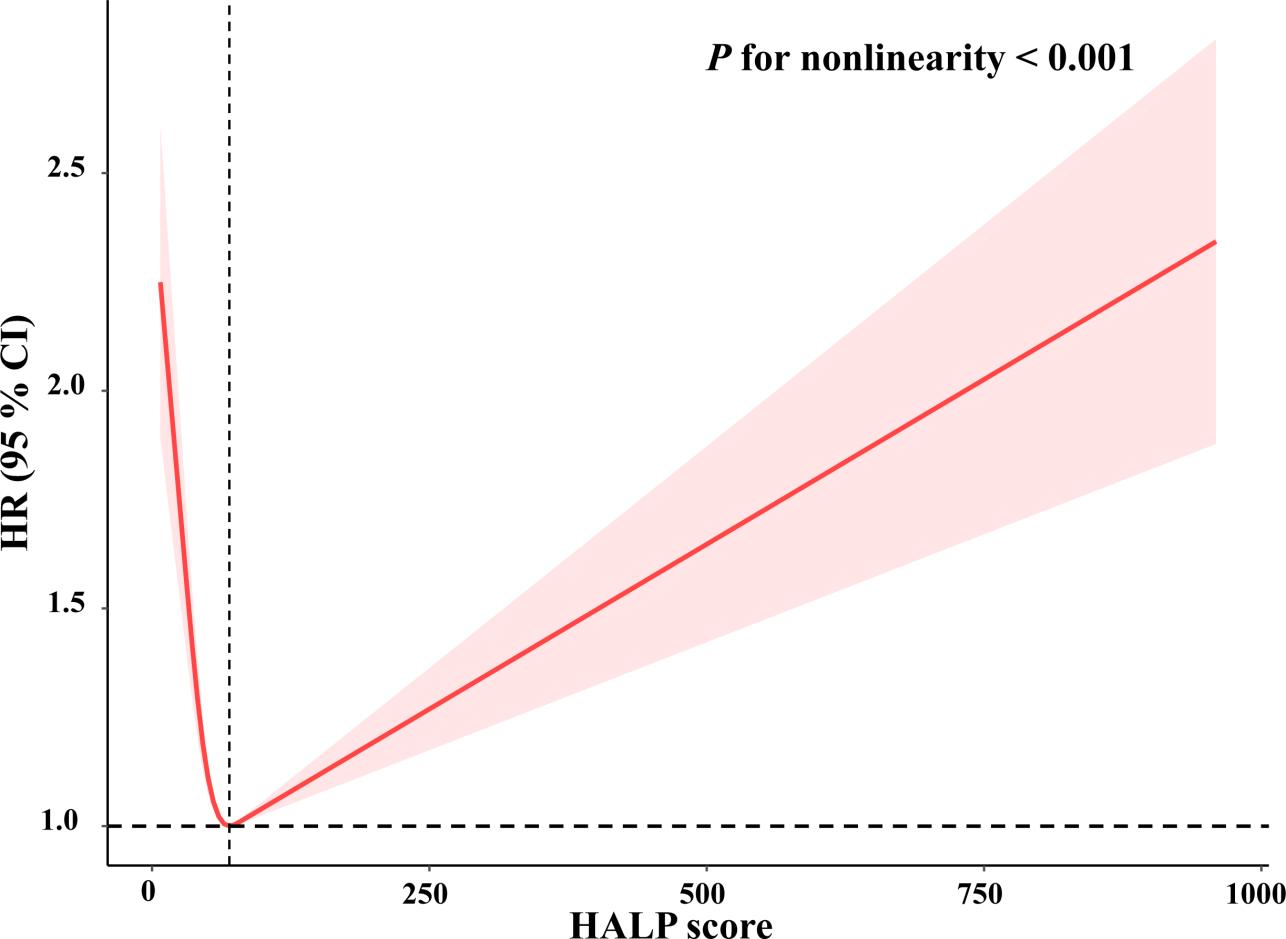
Restricted cubic spline plots of relationships between HALP score with all-cause mortality in the study population. Multivariable-adjusted HRs (red lines) and 95% CI (pink areas) for risk of mortality in model 3. Value of 74 was set as reference (vertical dashed line) for HALP score to predict risk of all-cause mortality.

**Figure 3:**
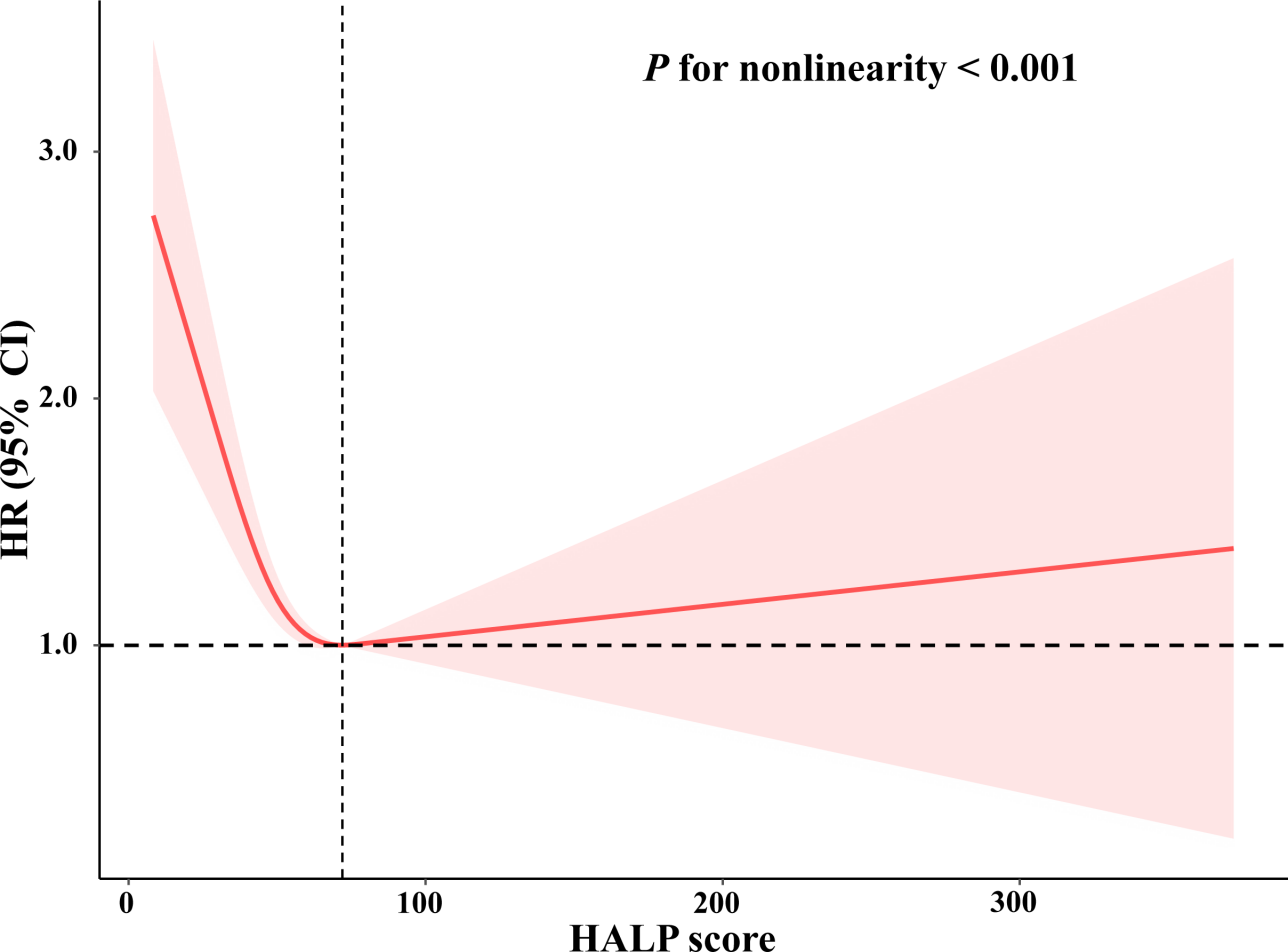
Restricted cubic spline plots of relationships between HALP score with cardiovascular mortality in the study population. Multivariable-adjusted HRs (red lines) and 95% CI (pink areas) for risk of mortality in model 3. Value of 72 was set as reference (vertical dashed line) for HALP score to predict risk of cardiovascular mortality.

As shown in **Tables 2** and **3**, three models of Cox proportional hazard regression all revealed that HALP scores were independently associated with long-term all-cause mortality and cardiovascular mortality. After adjusting for all confounders, compared with participants with low HALP scores, participants with highest HALP score had a lower risk of all-cause mortality (adjusted HR:0.80, 95% CI: 0.73, 0.89, *P* < 0.0001) and cardiovascular mortality (adjusted HR:0.61, 95% CI: 0.50, 0.75, p <0.0001), and mediate HALP score had the lowest risk of all-cause mortality (adjusted HR:0.68, 95% CI: 0.62, 0.75, *P* < 0.0001) and cardiovascular mortality (adjusted HR:0.60, 95% CI: 0.48, 0.75, *P* < 0.0001). In addition, all the trends of the classifications of tertiles were statistically significant (*P* for trend < 0.05). The restricted cubic spline curve revealed that HALP score was non-linearly and U-shaped correlated with all-cause mortality (*P* for non-linearity <0.001) (**Figure 2**) and cardiovascular mortality (*P* for non-linearity <0.001) (**Figure 3**).

**Table 2:** Cox regression analysis between HALP score and long-term mortality.

Outcomes	Model 1	*P value*	Model 2	*P value*	Model 3	*P value*
All-cause mortality						
HALP score						
Tertile 1	Reference		Reference		Reference	
Tertile 2	0.61 (0.56,0.67)	<0.0001	0.69 (0.63,0.76)	<0.0001	0.68 (0.62,0.75)	<0.0001
Tertile 3	0.75 (0.67,0.83)	<0.0001	0.85 (0.77,0.93)	<0.001	0.80 (0.73,0.89)	<0.0001
*P* for trend		<0.0001		<0.0001		<0.0001
Cardiovascular mortality						
HALP score						
Tertile 1	Reference		Reference		Reference	
Tertile 2	0.55 (0.44,0.69)	<0.0001	0.63 (0.51,0.77)	<0.0001	0.60 (0.48,0.75)	<0.0001
Tertile 3	0.61 (0.50,0.75)	<0.0001	0.68 (0.56,0.82)	<0.0001	0.61 (0.50,0.75)	<0.0001
*P* for trend		<0.0001		<0.0001		<0.0001
Cerebrovascular Mortality						
HALP score						
Tertile 1	Reference		Reference		Reference	
Tertile 2	0.98 (0.61,1.57)	0.940	1.20 (0.75,1.90)	0.450	1.18 (0.74,1.87)	0.480
Tertile 3	1.04 (0.62,1.73)	0.890	1.40 (0.85,2.33)	0.190	1.35 (0.81,2.26)	0.250
*P* for trend		0.893		0.188		0.248

Model 1 did not adjust for any confounding factors. Model 2 adjusted for age, race, education, sex, smoking status. Model 3 adjusted for all covariates in **Table 1** including age, sex race, education status, smoking status, diabetes mellitus, hypertension, hyperlipidemia, body mass index (BMI), hemoglobin (HB), lymphocyte (Lym), platelet (PLT), total cholesterol (TC), triglycerides (TG), low-Density Lipoprotein Cholesterol (LDL-C), high-Density Lipoprotein Cholesterol (HDL-C), and glycated hemoglobin (HbA1c).

**Table 3:** Subgroup analysis of HALP score and all-cause mortality.

	Tertile 1	Tertile 2	Tertile 3	*P* for interaction
All-cause mortality				
Age				0.135
<60	Reference	0.63 (0.49,0.82)***	0.75 (0.58,0.98)*	
>=60	Reference	0.67 (0.60,0.75) ***	0.70 (0.62,0.79) ***	
Sex				0.122
Male	Reference	0.75 (0.66,0.85)***	0.85 (0.74,0.97)**	
Female	Reference	0.73 (0.54,0.99)*	0.82 (0.62,1.09)	
Race				0.086
White	Reference	0.66 (0.59,0.74)***	0.82 (0.73,0.93)**	
Black	Reference	0.67 (0.55,0.82)**	0.83 (0.66,1.04)	
Mexican	Reference	0.73 (0.52,1.01)*	0.86 (0.63,1.16)	
Other	Reference	0.65 (0.40,1.06)	0.50 (0.36,0.69)***	
Smoke				0.237
Former	Reference	0.66 (0.57,0.77)	0.85 (0.73,1.00)	
Never	Reference	0.72 (0.62,0.85)***	0.70 (0.58,0.83)***	
Now	Reference	0.71 (0.58,0.88)**	0.84 (0.66,1.07)	
Education				0.251
<9th	Reference	0.72 (0.58,0.91)**	0.81 (0.64,1.03)	
9-11th	Reference	0.49 (0.40,0.61)***	0.67 (0.51,0.88)**	
High school	Reference	0.78 (0.66,0.92)**	0.87 (0.72,1.05)	
College	Reference	0.64 (0.54,0.77)***	0.84 (0.72,0.98)*	
Hypertension				0.006
Yes	Reference	0.69 (0.62,0.77)***	0.75 (0.67,0.85)***	
No	Reference	0.65 (0.53,0.79)***	0.94 (0.77,1.14)	
DM				0.031
Yes	Reference	0.57 (0.48,0.68)***	0.70 (0.58,0.84)***	
No	Reference	0.73 (0.65,0.81)***	0.88 (0.78,0.99)*	
Hyperlipidemia				0.409
Yes	Reference	0.71 (0.64,0.78)***	0.83 (0.74,0.93)**	
No	Reference	0.63 (0.48,0.81)***	0.69 (0.55,0.87)***	
BMI				0.707
<25	Reference	0.65 (0.55,0.76)***	0.77 (0.63,0.94)*	
25-29	Reference	0.79 (0.66,0.95)*	0.87 (0.72,1.05)	
>=30	Reference	0.68 (0.56,0.82)***	0.85 (0.70,1.02)	

‘***’: P < 0.001; ‘**’: P < 0.01; ‘*’: P < 0.05.

As described in **Table 4**, after adjusting for confounders in all models, there was no significant association between HALP score and risk of cerebrovascular mortality, and all trends of classifications of tertiles remained no significance (*P* for trend > 0.05).

**Table 4:** Subgroup analysis of HALP score and cardiovascular mortality.

	Tertile 1	Tertile 2	Tertile 3	*P* for interaction
Cardiovascular disease mortality				
Age				0.675
<60	Reference	0.54 (0.30,0.98)*	0.47 (0.26,0.84)*	
>=60	Reference	0.58 (0.47,0.72)***	0.55 (0.44,0.70)***	
Sex				0.424
Male	Reference	0.60 (0.44,0.81)***	0.62 (0.47,0.81)***	
Female	Reference	0.80 (0.59,1.08)	0.64 (0.48,0.86)**	
Race				0.186
White	Reference	0.58 (0.45,0.76)***	0.62 (0.48,0.80)**	
Black	Reference	0.66 (0.44,1.00)*	0.65 (0.42,1.01)	
Mexican	Reference	0.96 (0.54,1.70)	0.89 (0.49,1.59)	
Other	Reference	0.51 (0.20,1.33)	0.25 (0.08,0.74) *	
Smoke				0.684
Former	Reference	0.59 (0.43,0.79) **	0.62 (0.44,0.87) *	
Never	Reference	0.66 (0.47,0.92) *	0.55 (0.39,0.78) ***	
Now	Reference	0.61 (0.37,0.99)*	0.70 (0.41,1.19)	
Education				0.318
<9th	Reference	0.50 (0.31,0.79)**	0.43 (0.24,0.75)**	
9-11th	Reference	0.45 (0.27,0.75)**	0.74 (0.46,1.19)	
High school	Reference	0.71 (0.49,1.03)	0.82 (0.55,1.24)	
College	Reference	0.53 (0.36,0.80)**	0.53 (0.37,0.78)**	
Hypertension				0.713
Yes	Reference	0.58 (0.47,0.72)***	0.55 (0.44,0.69) ***	
No	Reference	0.65 (0.42,1.02)	0.65 (0.42,1.00)*	
DM				0.434
Yes	Reference	0.50 (0.34,0.72) ***	0.53 (0.38,0.72) ***	
No	Reference	0.64 (0.49,0.85)**	0.70 (0.54,0.90)*	
Hyperlipidemia				0.237
Yes	Reference	0.64 (0.51,0.80)***	0.65 (0.52,0.81)**	
No	Reference	0.36 (0.19,0.68)**	0.49 (0.28,0.86)*	
BMI				0.177
<25	Reference	0.64 (0.45,0.91)*	0.59 (0.40,0.88)*	
25-29	Reference	0.75 (0.53,1.07)	0.80 (0.56,1.16)	
>=30	Reference	0.47 (0.32,0.67)***	0.54 (0.39,0.76)**	

‘***’: P < 0.001; ‘**’: P < 0.01; ‘*’: P < 0.05.

### Subgroup analysis

Subgroup analysis was applied to evaluate any potential heterogeneity between HALP score and risk of cardiovascular or all-cause mortality. We found there were significant interactions between HALP score and hypertension (*P* for interaction = 0.006), and diabetes (*P* for interaction = 0.031) after adjusting all confounders (**Table 3**). Both participants with hypertension or diabetes with higher HALP score had higher risks of all-cause mortality. In addition, there was no significant interaction between HALP score and other covariates for risk of cardiovascular mortality (**Table 4**).

## Discussion

Atherosclerosis, the accumulation of fibrofatty lesions in the innermost layer of arteries, is regarded as one of the main pathogenic mechanisms of CCDs (1). Lymphocytes, consisting of differential subpopulations, play a crucial role in the initiation and progression of the pathogenesis of atherosclerosis via the activation of B and T lymphocytes (14). Platelets, produced by bone marrow megakaryocytes, serve as significant cells in the process of hemostasis and the formation of atheroma complications (15). Previous studies demonstrated that the combination indicator of platelet to lymphocyte ratio (PLR) was well-studied to predict mortality in all-cause and specific mortality (16–18). In addition, a meta-analysis of 11 cohorts with 12,619 patients showed higher PLR could predict the prognosis in myocardial infarction patients (19).

Malnutrition results in impaired physical and mental function and causes adverse clinical outcomes of disease due to insufficient intake or uptake of nutrition (20). Albumin, accounting for 50% of plasma proteins, is the most copious protein in the blood (21). Recently, despite there are different opinions concerning role of albumin in nutrition (22, 23), it is still considered as a marker of impaired nutritional status. Previous studies demonstrated that decreased levels of serum albumin were associated with morbidity and mortality in hospitalized patients (7, 24).

Anemia is caused by reductions in hemoglobin concentrations, red-cell counts or packed-cell volume, and impairs the oxygen demands of tissues (25). A systemic review indicated that anemia was correlated with adverse outcomes in cancer patients (26). In addition, accumulating evidence also demonstrated anemia was associated with all-cause and cardiovascular mortality (27–29).

HALP score, calculated based on the hemoglobin, albumin, lymphocyte, and platelet levels, could reflect the combination of inflammation and nutritional status. Convincing data indicated that the HALP score could predict the prognosis in various types of cancer patients, such as colorectal cancer (30), gastric cancer (31), cervical cancer (32), and lung cancer (33). However, limited information exists on the association between HALP score and all-cause and specific mortality. Given the established role of HALP score in the prognosis of cancer patients, we explored and demonstrated that HALP score was independently associated with long-term cardiovascular and all-cause mortality but not cerebrovascular mortality. Further analyses showed that the HALP score had a non-linear association with cardiovascular and all-cause mortality.

In subgroup analysis, the association displayed more profound in groups with hypertension or diabetes for all-cause mortality. Hypertension and diabetes are the two most common comorbidities, which correlate with the dysfunction of endothelial cells and the process of oxidative stress, inflammation, and atherosclerosis (34–37). Mounting evidence illustrated that hypertension and hyperglycemia are the two independent indicators of worst outcomes. Pasquale et al. further demonstrated that hyperglycemia could aggravate cognitive and physical impairment in hypertensive older adults (38, 39). However, the association was not profound in groups with hypertension or diabetes for cardiovascular mortality. The possible explanation may be that the number of participants who suffered from cardiovascular mortality was relatively small. Hence, research concerning HALP score and cardiovascular mortality in hypertension or hyperglycemia is warranted.

In addition, our study found a non-linear association between HALP score and all-cause mortality or cardiovascular mortality. The possible reasons may be as follows: on the one hand, a cohort study of 170,078 men and 122,116 women without cardiovascular disease demonstrated that low or high levels of hemoglobin were associated with elevated cardiovascular and all-cause mortality (40). On the other hand, a cohort study of 21,252 adults demonstrated a U-shaped relationship between platelet count and mortality, in which platelet <175 × 10^9^/L or >300 × 10^9^/L was significantly increased for mortality (41). Tsai M et al. found that both thrombocytopenia and increased platelet count were related to cardiovascular mortality (42). Therefore, the possible explanation may be that levels of hemoglobin or platelets were higher or lower among participants in this cohort. More research is warranted to determine the role of HALP score in cardiovascular and all-cause mortality.

A study of 1,337 stroke patients demonstrated that the HALP score could forecast stroke recurrence and mortality within 90 days and 1 year (11). In contrast, our study did not ascertain an association between HALP score and cerebrovascular mortality. The discrepancy may be explained in part by the fact that number of participants suffered from cerebrovascular mortality was too small so that statistical significance was not obvious. Therefore, further clinical investigations are needed to evaluate whether HALP score was correlated with cerebrovascular mortality.

Our study had several strengths. Firstly, we evaluated the interaction from a prominent representative and large-scale sample. Secondly, three Cox proportional hazard regression models were further to substantiate the relationship between HALP score and cerebrovascular, cardiovascular, and all-cause mortality. Last, this study also investigated the exact shape of the association of HALP score and cardiovascular and all-cause mortality through restricted cubic spline curves. However, the conclusions should be interpreted with caution. On the one hand, although we adjusted the potential confounding factors as far as possible, we could not completely exclude the influence of other possible covariates. On the other hand, our sample was extracted from the U.S. database, hence it may not generalize to other populations. Taken together, more studies are needed to further investigate the effect of HALP score in clinical practice.

## Conclusion

In summary, our study demonstrated that HALP score was independently associated with risk of cardiovascular and all-cause mortality, but not cerebrovascular mortality in the general population, which indicated that HALP score might be a reliable predictor of long-term outcomes.

## Data availability statement

The original contributions presented in the study are included in the article/supplementary materials, further inquiries can be directed to the corresponding author/s.

## Ethics statement

The studies involving human participants were reviewed and approved by NCHS Ethics Review Board. Written informed consent to participate in this study was provided by the participants’ legal guardian/next of kin. Written informed consent was obtained from the individual(s) for the publication of any potentially identifiable images or data included in this article.

## Author contributions

HP: data analysis and writing—original draft. SL: formal analysis and writing—reviewing and editing original draft. All authors contributed to the article and approved the submitted version.
